# Preparation and Properties of Waste Corrugated Paper Fiber/Polylactic Acid Co-Extruded Composite

**DOI:** 10.3390/polym14214569

**Published:** 2022-10-28

**Authors:** Jian Su, Mannan Yang, Xiaomei Zhang, Changqing Fang, Yamin Zheng, Lu Pei, Ming Liu

**Affiliations:** 1 Faculty of Printing, Packaging Engineering and Digital Media Technology, Xi’an University of Technology, Xi’an 710054, China; 2 School of Mechanical and Precision Instrument Engineering, Xi’an University of Technology, Xi’an 710048, China; 3 Jiangsu Weixing New Materials Co., Ltd., Yangzhou 225600, China

**Keywords:** waste corrugated paper fiber, polylactic acid, co-extrusion, surface modification, reinforce

## Abstract

In order to explore the methods of recycling waste paper, reduce environment pollution, and develop a circular economy, the application of waste corrugated paper to the strengthening of polylactic acid (PLA) was studied. Plant fiber from waste corrugated paper (WCPF) was used to prepare WCPF/PLA composite via co-extrusion. The WCPF was extracted from the waste corrugated paper by beating in a Valli beating machine and grinding in a disc grinder. KH-550 coupling agent was used to modify the surface of WCPF to improve the interface adhesive strength between the WCPF and PLA matrix. The effects of the contents of WCPF and KH-550 coupling agent on the mechanical properties, microstructure, crystallization properties, and thermostability of the WCPF/PLA composite were studied. The results show that the WCPF can be well separated from each other. The WCPF can be uniformly dispersed in the PLA matrix through a co-extrusion process. WCPF can increase the mechanical strength and deformation resistance ability of WCPF/PLA composite, and KH-550 coupling agent can further improve that of the WCPF/PLA composite. This study is of obvious significance to the recycling of waste paper and the development of a circular economy.

## 1. Introduction

Paper is widely used in people’s daily lives, and it comes from a wide range of renewable plant sources. The main composition of paper is plant fiber which is mainly composed of natural polymers, such as lignin, hemicellulose, cellulose, etc. The output of waste paper is huge every year, but it is also a potential resource. The recycle of waste paper is of great significance to environmental protection and circular economy [1].

The reinforcement of polymer by filling with plant fiber materials is a common reinforcement method [2]. Plant fiber is one of the most abundant natural materials in nature. It can come from wood (softwoods and hardwoods) or non-wood (seed, fruit, bast, leaf, stalk, grass, etc.) materials [3]. The plant fiber has many advantages such as wide source, low price, complete degradation, etc., and it also has a high strength to weight ratio [4]. Although the mechanical properties of plant fiber are much lower than the most widely used synthetic fibers, the high property to density ratio, low density, high stiffness, and high strength make it comparable to the values of the synthetic fibers [5].

The plant fiber can be obtained from a variety of plants by mechanical, biological processes or chemical methods, as well as combined processes. Yao et al. [6] studied the effects of the surface modification of reed fiber on the mechanical properties, crystallization, and thermostability of PLA composite. The cut reed stems were treated with glacial acetic acid and hydrogen peroxide mixed solution first, then screened, freeze-dried, and treated with sodium hydroxide solution, and freeze-dried again after cleaned to produce unmodified natural plant fibers.

As a biodegradable and environmentally friendly plastic, PLA has a wide range of raw materials sources, good biocompatibility, and processing performance, and has a wide range of application potential in many fields [7]. It is one of the most promising polymer materials. However, its disadvantages of high brittleness and high cost also greatly limit its application. Therefore, PLA usually needs to be enhanced to improve the application performance [8]. Plant fiber is a kind of biodegradable material and can be used to prepare a fully biodegradable composite material with the biodegradable PLA. Plant fibers derived from a variety of plants have been used to enhance PLA, such as flax, kenaf, straws, jute, cotton, wood, etc. [9,10]. Yang et al. [11] prepared ramie fiber/PLA composite with optimized mechanical properties through the combination of two-dimensional braiding and three-dimensional weaving method. Porras et al. [12] used natural plant fiber fabric to strengthen PLA. They made natural plant fiber and PLA thin sections, then pressed three layers of PLA with two layers of natural fiber to prepare PLA-based composite materials. The results show that natural plant fiber can improve the mechanical properties of PLA.

It is well known that paper is made of plant fiber which is rich in hydroxyl groups and bonded by hydrogen bonds. In the process of papermaking, the colloid, intercellular substance and part of the lignin in the raw plant materials have been removed, and the fiber in the paper is a relatively pure single bundle of plant fiber [1]. Therefore, it is much easier and simpler to obtain plant fiber from waste paper than from raw plant materials, hence the waste paper as raw material to reinforce PLA has good research prospects. At present, it has been reported that office waste paper and waste newspaper can be used as raw materials to prepare PLA composite. However, there is a large amount of hydroxyl groups on the surface of paper fiber, which can easily cause agglomeration. Zhang et al. [13] directly smashed the waste paper or smashed it after deinking by a high-speed pulverizer. However, the blade of the pulverizer can cut through the fibers and the plant fibers in waste paper are difficult to separate thoroughly from each other. Tao et al. [14] grinded the waste office paper with a ball mill machine, but the result showed that the length to diameter ratio of paper fiber is greatly reduced. Therefore, the pretreatment process of waste paper still needs to be optimized.

Interface adhesive strength between the plant fiber and polymer matrix plays an important role in deciding the mechanical performance of the composite. The main components of plant fiber include lignin, hemicellulose, cellulose, a small amount of pectin, waxes, and other low-molecule substances, which contain lots of hydrophilic hydroxyl groups. Therefore, the incorporation of plant fiber in hydrophobic and non-polar polymers matrix leads to a limited performance improvement [15]. The surface modification, including physical treatments and chemical treatments, of plant fiber is an important method to improve its interfacial adhesion with polymer [16,17]. Among the various chemical treatment methods, the surface treatment of plant fiber with silane coupling agent is a common method to enhance the interfacial adhesion between the plant fiber and PLA [18]. A typical silane coupling agent has two reactive groups, one end of silane agent with alkoxysilane groups can react with hydroxyl, the other end can interact with the polymer matrix [19]. Qian et al. [20] used (3-mercaptopropyl) trimethoxy silane (A-189) to modify the bamboo cellulose nano whiskers and the surface-modified bamboo cellulose nano whiskers were used to toughen the brittle PLA matrix. The result showed that the coupling agent was grafted on the surface of cellulose via Si-O-C covalent bonds and hydrogen bonding. Hydrogen bonding can be generated between the coupling agent and PLA backbone. Liu et al. [21] treated corn stalk fiber with amino-propyl-triethoxysilane. The results showed that silane treatment can promote better mechanical interlocking by improving the interfacial adhesion.

In this work, the WCPF is extracted from the waste corrugated paper by beating and grinding which would not destroy the fiber structure, and the fibers can separate well from each other. The KH-550 silane coupling agent was used to modify the surface of WCPF. The WCPF and PLA were blended by a twin screw extruder. This study provides a new idea for the recycling of waste paper and an optimization method for the technology of plant fiber reinforced PLA composite.

## 2. Materials and Methods

### 2.1. Materials

Polylactic acid (the density is about 1.246 g/cm^3^, the melt flow index is about 8 g/10 min (190 °C, 2.16 kg), and it is 100 mesh powder) used in this study was from Dongguan Yingsheng Plastic Chemical Co., Ltd. The corrugated paper came from waste corrugated boxes used for express delivery. The contents of the prepared WCPF are cellulose (13.2 wt%), hemicellulose (18.7 wt%), lignin (44.0 wt%), inorganic salt (8.0 wt%), and others (16.1 wt%) which were determined via the Van Soest detergent method [22]. The ethylenediaminetetraacetic acid disodium salt (C_10_H_14_N_2_Na_2_O_8_), sodium borate (Na_2_B_4_O_7_), ethylene glycol ether (C_4_H_10_O_2_), disodium hydrogen phosphate (NaH_2_PO_4_), cetane trimethyl ammonium bromide (C_19_H_42_BrN), and sodium alkyl sulfate (C_12_H_25_SO_4_Na) used for the Van Soest detergent were from Nanjing Reagent Co., Ltd. (Nanjing, Jiangsu Province, China), and were analytically pure. Absolute ethanol (C_2_H_6_O, analytically pure) was from Shanghai Yinxiang Biotechnology Co., Ltd. (Shanghai, China). Silane coupling agent (KH-550) was from Nanjing Yudeheng coupling agent Factory (Nanjing, China).

### 2.2. Preparation of WCPF/PLA Composite

**Figure 1:**
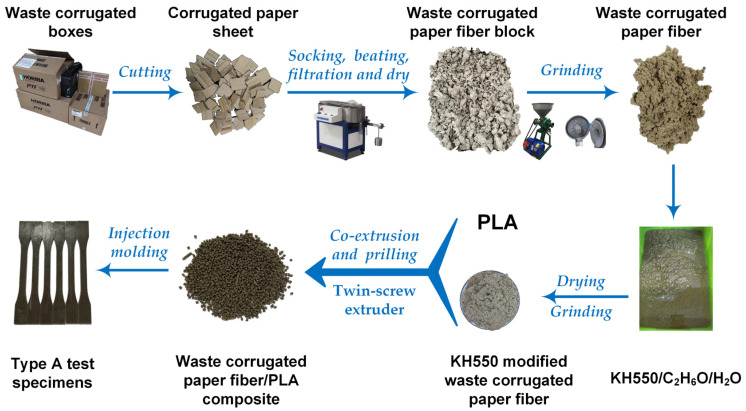
Preparation process diagram of the WCPF/PLA composite.

The preparation flow chart of WCPF/PLA composite is shown in Figure 1. Firstly, the attachments on the surface of the corrugated box were removed, then the corrugated paper was cut into 2 cm × 2 cm pieces of sheets. About 600 g of the paper sheets were dipped in water at room temperature for 24 h, then the soaked paper sheets were pulped for 30 min by an IMT-VL01 Valli beating machine (Dongguan International material Tester Precision Instrument Co., Ltd., Dongguan, China). The waste paper pulp was washed by water and filtered to obtain the paper fiber block, then fiber block was dried at 80 °C for 12 h, which was grinded by a disc grinding machine (SX150, Hubei Province Shishou City Sixin Machinery Factory, Shishou, China) and became shaggy and well separated paper fiber. A certain amount of KH-550 was added into 2.1 L of the solution with a mass ratio of water to absolute ethanol of 1:19. 250 g of the WCPF was soaked in the above solution for 24 h. The solution was filtered out by filter cloth to obtain the WCPF blocks, which were dried at 80 °C for 12 h and then ground again. A twin screw extruder (SHJ-35, Nanjing Hanyi Mechanical and Electrical Co., Ltd., Nanjing, China) was used to prepare the WCPF/PLA composite. The twin screw extruder used in this work was a co-rotating intermeshing twin screw extruder with two parallel screws. The length–diameter ratio of the screw is 50 with a diameter of 35.6 mm. The temperature of the extrusion die mouth was set as 163 °C, and the temperatures of the other 8 heating areas were 130 °C, 140 °C, 145 °C, 150 °C, 155 °C, 160 °C, and 160 °C, respectively. The rotation speed of the screw was 25 rpm, with a raw material feed speed of about 10 kg/h. Before feeding the raw materials, the extruder was preheated for 1 h. The WCPF and PLA were artificial premixed. The extruded WCPF/PLA composite strips were cut into particles by a granulator and then the WCPF/PLA composite particle was dried at 60 °C for 1 h. The preparation conditions of the WCPF/PLA composite samples are listed in Table 1.

### 2.3. Characterization

The test specimens used for mechanical property test were prepared by injection molding using a TY-7003 horizontal injection molding machine (Jiangsu Tianyuan Testing Equipment Co., Ltd., Yangzhou, China). The test specimens were type A multipurpose specimens (ISO 3167). The injection molding machine have three heating area, and the temperature of each heating area was 140 °C, 150 °C, and 160 °C, respectively. In addition, the specimen mold was not heated. The injection time was 5 s with a 57 MPa injection pressure. The pressure was held for 15 s, and after 15 s of cooling, the test specimens were obtained. A HT-2402 universal testing machine (Hongda Instrument Co., Ltd., Taizhong, China) with a 10 kN sensor was used to test the mechanical properties of the WCPF/PLA composite. The tensile strength test was carried out according to ISO 527-2 standard with a 5 mm/min tensile speed and the clamping distance was 115 mm. The flexural strength test was carried out according to ISO 178 standard with a 5 mm/min pressing speed using three-point flexural method and the span was 64 mm. The test results were the average of three measurements (test 5 times, removing a maximum and a minimum value) and the standard deviations are shown in the results. The microstructure of the WCPF/PLA composite was analyzed on a JSM-6390 scanning electron microscopy (SEM, JEOL, Akishima, Japan) and the acceleration voltage was 15 kV. The samples were broken off after freezing in liquid nitrogen. A gold spraying process was carried out before analysis.

A 200F3 differential scanning calorimeter (DSC, NETZSCH-Gerätebau GmbH, Selb, Germany) was used to analyze the thermal behavior of WCPF/PLA composite. In order to eliminate the thermal history, the sample was heated from 20 °C to 150 °C at the heating rate of 10 °C/min firstly. Next, the sample was cooled to 20 °C at the cooling rate of 10 °C/min. Finally, the sample was heated from 20 °C to 220 °C at the heating rate of 10 °C/min. The whole test was carried out under the N_2_ atmosphere (50 mL/min) and 10 mg of the sample was used for the DSC test. 

A 209F3 thermogravimetric analyzer (TG, NETZSCH-Gerätebau GmbH, Selb, Germany) was used to analyze the thermal decomposition behavior of the WCPF/PLA composite. The test was carried out from 30 °C to 600 °C at the heating rate of 10 °C/min. The whole test was carried out under N_2_ atmosphere (50 mL/min) and 10 mg of the sample was used for the TG test.

An XRD-7000 X-ray diffraction apparatus (XRD, Shimadzu, Kyoto, Japan) was used to analyze the crystallization of the WCPF/PLA composite. The test was carried out from 10 to 60° with 10°/min scanning speed and 0.02° sampling pitch. The samples were prepared into thick sheets for the XRD test. The X-ray diffraction apparatus using a Cu target, Kα1 line, Ni filter, 50 mA current, and 40 kV voltage.

## 3. Results and Discussion

### 3.1. Mechanical Properties of WCPF/PLA Composite

The mechanical performance parameters of PLA and WCPF/PLA composite are listed in Table 2. The tensile strength and flexural strength of PLA are 27.2 ± 0.8 MPa and 33.7 ± 1.4 MPa, respectively. The tensile properties of the WCPF/PLA composite with a small amount of WCPF have a slight decrease when the WCPF content is 5 wt%. This may be due to the degradation of PLA in the co-extrusion process and the internal defects of the WCPF/PLA composite produced during extrusion and injection molding, and a small amount of WCPF is not enough to offset the performance loss caused by the above adverse factors, resulting in a decrease in tensile properties. When the WCPF content is more than 20 wt%, the tensile properties of the WCPF/PLA composite have obvious improvement. This is because when the samples receive external pull force, the force can transfer to the WCPF through interfacial bonding force between WCPF and PLA, and there is enough WCPF to bear the tension force, thus improving the tensile strength of the material. When the content of WCPF is 30 wt%, the tensile strength reaches the maximum value. When more WCPF was used without other additives, the extruded composite strip broke easily, and it was difficult to achieve continuous extrusion.

In addition, when the WCPF content is 30 wt%, the tensile strength of the WCPF/PLA composite can be obviously improved after adding KH-550 coupling agent, which indicates that KH-550 can further improve the mechanical properties of the WCPF/PLA composite by enhancing the interface bonding force between WCPF and PLA matrix. When the sample is subjected to tension force, the microcrack first occurs in the PLA matrix, and the WCPF can prevent the crack from expanding and improve the tensile strength of the WCPF/PLA composite through the interface bonding force between the WCPF and the PLA matrix.

The flexural strength of all WCPF/PLA composite improves to a certain extent. In addition, the flexural strength of WCPF/PLA composite can be obviously further improved by increasing the use of KH-550 coupling agent. Only 0.3 wt% of the KH-550 coupling agent can get a good strengthening effect, and continue to increase the amount of KH-550 coupling agent, the flexural strength increase amplitude is not obvious. When the WCPF/PLA composite is subjected to bending and cracking, the internal filled WCPF can prevent the further expansion of the crack through the interface bonding force between it and PLA matrix, and bear part of the bending force to improve the bending resistance of the WCPF/PLA composite. In general, an appropriate amount of coupling agent can improve the mechanical properties of the WCPF/PLA composites. Too much coupling agent will reduce the mechanical properties of the WCPF/PLA composites. The additional amount of 0.3 wt% KH-550 achieved the best enhancement effect and the mechanical properties of the WCPF/PLA composites decreased with the increase of the amount of KH-550. The relatively low tensile strength decreases for P30-6, P30-9 samples may be because of the errors that due to internal defects of the WCPF/PLA composites. WCPF can obviously reduce the elongation at break of WCPF/PLA composite, and the more WCPF used, the lower the elongation at break because the WCPF can limit the movement of the PLA molecular chain, and even causing stress concentration which leads to decrease of the elongation at breaking of the WCPF/PLA composite. Moreover, the WCPF can be easily oriented in the PLA melt due to its large length-to-diameter ratio, and it can affect the orientation of the PLA molecular chain. In addition, the WCPF can cause internal defects such as impurities voids, gaps, or cracks, and these defects can easily cause stress concentration, thus reduce the elongation at breaking [2]. However, the KH-550 coupling agent can enhance the interface adhesive strength between WCPF and PLA and reduce the internal defects of WCPF/PLA composite to improve the toughness, thus increase the elongation at break. 

The tensile strength and flexural strength of the WCPF/PLA composite increase, while the elongation at break decreases. Therefore, the tensile modulus and flexural modulus of the WCPF/PLA composite increased to different degrees, indicating that the anti-deformation ability of the WCPF/PLA composite was enhanced. The use of KH-550 improved the elongation at break of the WCPF/PLA composite; it also obviously improved the tensile strength and flexural strength of the WCPF/PLA composite, so it can still further improve the anti-deformation ability of the material.

### 3.2. Microstructure of WCPF/PLA Composite

**Figure 2:**
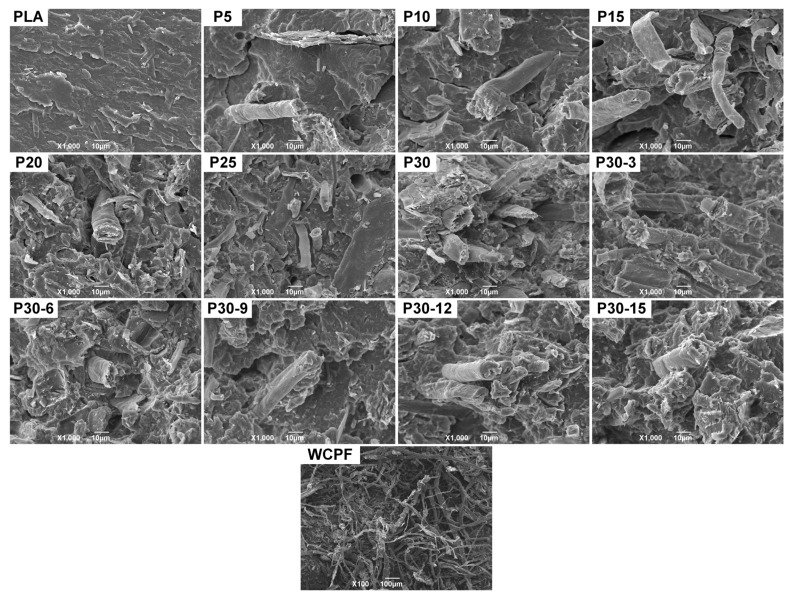
Microstructure of WCPF, PLA, and WCPF/PLA composite.

Figure 2 shows the fracture surface microscopic morphology of PLA, WCPF/PLA composite, and WCPF. The result shows that the WCPF is a kind of flat strip plant fiber with a high length-diameter ratio and the fibers are well separated from each other. This indicates that paper fibers can be well separated by the shearing and rubbing action in the process of beating and grinding. The width of the WCPF is about 10–60 μm. Although the full length of the WCPF is not totally shown in the visual field, it can be seen that the length of the WCFP is more than 2 mm in length. The WCPF is evenly dispersed in the PLA matrix, and there is no obvious agglomeration or intertwining of the WCPF. With the increase of the WCPF content, the distribution density of the WCPF in the field of view increases.

When the WCPF is not modified, some holes with a smooth wall can be found in the fracture section of the WCPF/PLA composite, and some long WCPF are pulled out from the PLA matrix. In addition, there are many cracks in the fracture section of the WCPF/PLA composite, while there are no such cracks in the fracture section of PLA, which indicates that the addition of WCPF destroys part of the structure of the PLA matrix and it could lead to stress concentration. In contrast, there are almost no smooth wall holes in the WCPF/PLA composite that the KH-550 modified WCPF is used. Only some shallow irregular pits are observed, which were formed by the pulling cut of WCPF. The surrounding PLA matrix that adheres to the WCPF was pulled out when the composite was stressed. Moreover, almost no long fibers were pulled out in the fracture section, which indicates that KH-550 coupling agent can obviously enhance the interface adhesive strength between WCPF and PLA matrix. The enhancement of interfacial binding force between WCPF and PLA matrix can further improve the mechanical properties of the WCPF/PLA composite. The result is consistent with the mechanical properties result. In addition, there are some very subtle holes and cracks in the section. These defects are caused by insufficient exhaust, etc., in the process of co-extrusion and injection molding.

### 3.3. DSC Analysis

**Figure 3:**
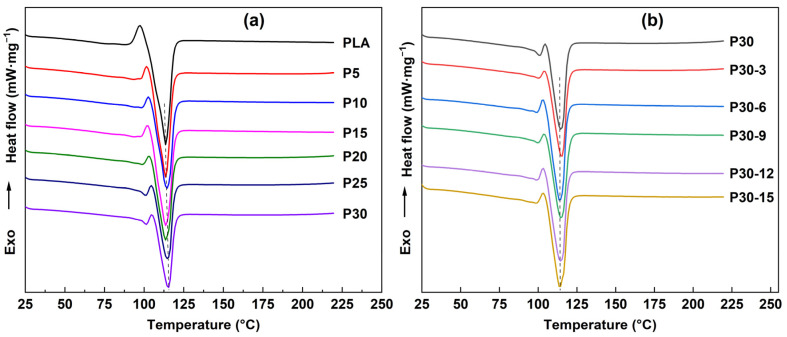
(**a**) DSC curves of PLA and WCPF/PLA composite with different contents of WCPF. (**b**) DSC curves of the WCPF/PLA composite with 30 wt% WCPF treated by different contents of KH-550 coupling agent.

Figure 3a shows the DSC curves of PLA and WCPF/PLA composite with different WCPF contents. Figure 3b shows the DSC curves of the WCPF/PLA composite with 30 wt% WCPF treated by different contents of KH-550 coupling agent. The melting temperature of the pure PLA is about 113.5 °C. The melting temperature difference of the WCPF/PLA composite material prepared in different conditions was not obvious, and it was also not obviously affected by the amount of the WCPF and KH-550 coupling agent. No chemical reaction occurred between WCPF and PLA, so no new material was generated. Therefore, the melting temperature of the WCPF/PLA composite did not change obviously. In addition, no obvious glass transition temperature peak was found in the DSC curves, which could be due to the high crystallinity of the purchased PLA [6,23].

### 3.4. TG Analysis

**Figure 4:**
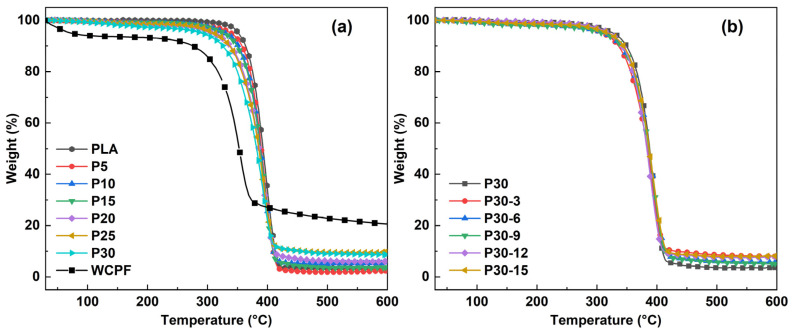
(**a**) TG curves of WCPF, PLA, and WCPF/PLA composite with different contents of WCPF. (**b**) TG curves of the WCPF/PLA composite with 30 wt% WCPF treated by different contents of KH-550 coupling agent.

Figure 4a shows the TG curves of WCPF, PLA, and WCPF/PLA composite with different contents of WCPF. According to the TG curves, the decomposition temperature of WCPF was significantly lower than that of PLA and WCPF/PLA composites. When the weight loss of PLA was 5 wt%, the corresponding temperature was about 350 °C. However, for the WCPF/PLA composite, the temperature corresponding to 5 wt% weight loss had a certain amount of decline, and the decrease trend became more obvious with the increase of the content of WCPF. This indicates that the initial decomposition temperature of the WCPF/PLA composite was reduced after the addition of WCPF. This is because the WCPF contains a large amount of lignin, cellulose, and hemicellulose, which easily to absorb moisture and have a relatively low thermal decomposition temperature. In the low temperature period, the volatilization of part of the water and a small amount of decomposition of lignin, cellulose, and hemicellulose reduced the thermal stability of the WCPF/PLA composite.

Figure 4b shows the TG curves of the WCPF/PLA composite with 30 wt% WCPF which was treated by different contents of KH-550 coupling agent. It can be seen that the TG curves of all samples had no obvious difference. Although the KH-550 coupling agent can improve the mechanical properties of the WCPF/PLA composite, it had little influence on the thermal stability of the WCPF/PLA composite. This is because there was no strong chemical bond between the KH-550, WCPF, and PLA. Moreover, the amount of KH-550 coupling agent was very small, which was not enough to obviously affect the thermal stability of the WCPF/PLA composite.

In general, the thermal decomposition of WCPF/PLA composite can be roughly divided into three stages. From 50 to 280 °C is the dehydration of WCPF/PLA composite and the decomposition of small molecules such as pectin, hemicellulose, etc. in WCPF; 280–420 °C is the decomposition of most of the PLA, cellulose, hemicellulose, and lignin in WCPF; and above 420 °C is the decomposition of the residual matter in the WCPF [24].

### 3.5. XRD Analysis

**Figure 5:**
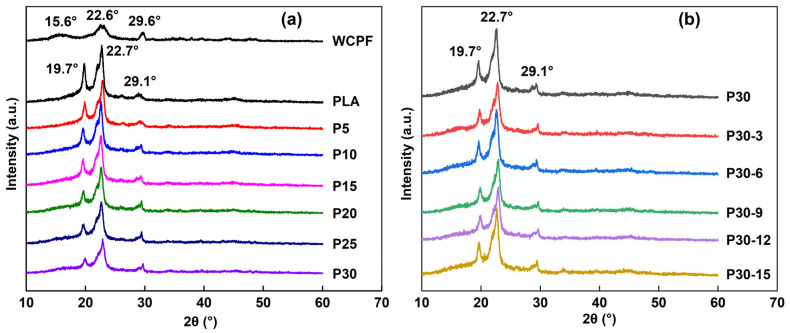
(**a**) XRD patterns of WCPF, PLA, and WCPF/PLA composite with different contents of WCPF. (**b**) XRD patterns of the WCPF/PLA composite with 30 wt% WCPF which treated by different contents of KH-550 coupling agent.

Figure 5a shows the XRD patterns of the WCPF, PLA, and WCPF/PLA composite with different contents of WCPF. As shown in Figure 5a, there are three diffraction peaks of WCPF. The diffraction peaks located at about 15.6° and 22.6° are correspond to the (101) and (002) crystal planes of the cellulose. The diffraction peak of WCPF located at about 29.6° is mainly due to some unknown material in the WCPF. There are three main characteristic diffraction peaks at about 19.7°, 22.7°, and 29.1° in the XRD patterns of PLA and WCPF/PLA composite, which correspond to the diffraction peaks of the α crystalline PLA [6]. No new diffraction peaks appear in the XRD patterns of WCPF/PLA composite, and the diffraction peak positions corresponding to PLA does not change obviously, which indicates that the addition of WCPF did not change the crystal form of PLA. However, the diffraction peak intensities of the WCPF/PLA composites slightly decreased compared with PLA, and the trend of decreasing was more obvious with the increase of WCPF content. This is because the diffraction peak position of WCPF was roughly the same as that of PLA, but the diffraction intensity of WCPF was lower than PLA. Therefore, the higher the content of WCPF, the lower the diffraction intensity of the WCPF/PLA composite will be.

Figure 5b shows the XRD patterns of the WCPF/PLA composite with 30 wt% WCPF treated by different contents of KH-550 coupling agent. With the increase of the amount of KH-550 coupling agent, the intensity of the diffraction peaks of the WCPF/PLA composite decreased slightly. This is because the KH-550 coupling agent grafted on the WCPF surface and enhanced the interfacial bonding force between the WCPF and PLA. The WCPF binds more closely to the PLA matrix, and with the increase of the amount of KH-550 coupling agent, the internal crystal structure of PLA was destroyed, which was manifested by the decrease of the intensity of the XRD peak.

## 4. Conclusions

Paper fibers can be well separated from each other by soaking, beating, and grinding, and the fibers can maintain a large length to diameter ratio. The WCPF can be evenly dispersed into the PLA matrix by co-extrusion. The addition of WCPF can improve the mechanical properties of PLA. KH-550 coupling agent can enhance the interface bonding force between WCPF and PLA matrix to further improve the mechanical properties of WCPF/PLA composite. The best enhancement effect is achieved when the content of WCPF is 30 wt%, and the amount of 0.3 wt% KH-550 coupling agent can obtain an obvious further enhancement effect. The addition of WCPF can slightly decrease the thermal stability of WCPF/PLA composite, and slightly reduce the crystallinity of PLA without changing the crystal type. This study provides a new method of waste paper recycling. The WCPF/PLA composite can be used for 3D printing or disposable packaging materials, such as the preparation of foaming material and some plastic components in the disposable packaging, etc. The biodegradable PLA can replace the non-degradable polyolefin materials, and the addition of waste paper fibers can reduce the use cost of PLA, which is of great significance to environmental protection and circular economy.

## Figures and Tables

**Table 1:** The raw material ratio of the WCPF/PLA composite.

Sample	WCPF Content (wt%)	Concentration of KH-550 Coupling Agent in Solution (wt%)
PLA	0	-
P5	5	-
P10	10	-
P15	15	-
P20	20	-
P25	25	-
P30	30	-
P30-3	30	0.3
P30-6	30	0.6
P30-9	30	0.9
P30-12	30	1.2
P30-15	30	1.5

**Table 2:** Mechanical properties of WCPF/PLA composites prepared in different conditions.

Samples	Tensile Strength (MPa)	Elongation at Break (%)	Tensile Modulus (MPa)	Flexural Strength (MPa)	Flexural Modulus (MPa)
PLA	27.2 ± 0.8	6.6 ± 0.3	769 ± 67	33.7 ± 1.4	898 ± 56
P5	27.1 ± 0.2	5.9 ± 0.2	848 ± 16	34.9 ± 1.1	1014 ± 66
P10	27.4 ± 0.4	5.6 ± 0.3	1084 ± 11	44.7 ± 2.9	1456 ± 32
P15	27.8 ± 0.4	5.2 ± 0.5	1230 ± 23	40.6 ± 2.4	1620 ± 12
P20	29.6 ± 0.4	4.8 ± 0.1	1510 ± 20	51.2 ± 3.4	2055 ± 78
P25	30.7 ± 0.5	3.9 ± 0.3	1804 ± 15	55.0 ± 2.7	2599 ± 50
P30	31.4 ± 0.5	3.8 ± 0.1	1913 ± 54	54.1 ± 1.4	2636 ± 71
P30-3	36.1 ± 0.5	4.3 ± 0.1	1914 ± 50	64.9 ± 1.8	2836 ± 23
P30-6	33.5 ± 0.4	4.9 ± 0.2	1606 ± 16	61.2 ± 3.5	2333 ± 46
P30-9	32.7 ± 1.3	3.9 ± 0.7	1648 ± 14	56.7 ± 3.1	2708 ± 61
P30-12	34.2 ± 0.3	4.9 ± 0.2	1611 ± 27	67.1 ± 1.9	2776 ± 56
P30-15	34.6 ± 0.8	4.8 ± 0.6	1641 ± 54	68.2 ± 3.1	2939 ± 48

## Data Availability

Data presented in this study are available on request from the corresponding author.
